# The combined effects of Tabata training and cinnamon supplementation on metabolic changes and body composition in soldiers with overweight or obesity

**DOI:** 10.1080/15502783.2025.2564237

**Published:** 2025-09-19

**Authors:** Reza Sabzevari Rad, Hamid Omidi, Milad Alipour

**Affiliations:** a Imam Ali Military University, Faculty of Command and Management, Department of Physical Education and Sport Sciences, Tehran, Iran; b University of Mohaghegh Ardabili, Department of Sport Physiology, Faculty of Educational Sciences and Psychology, Ardabil, Iran

**Keywords:** High-intensity interval training, nutritional supplements, metabolic health, body composition

## Abstract

**Aim:**

This study investigated the effect of the combining Tabata training and cinnamon supplementation on metabolic changes and body composition in overweight and obese soldiers.

**Materials and Methods:**

40 overweight and obese soldiers were divided into Tabata (T), Tabata training+supplement (T+S), supplement (S) and control (C) groups. The intervention completed during eight weeks with three sessions per week. Pre- and post-intervention assessments included body composition (body mass index [BMI], body fat percentage [BFP], performance parameters) push-up, squat, plank and vertical jump), metabolic markers (fasting blood sugar [FBS], insulin and [HOMA], liver enzymes (Serum Glutamic-Oxaloacetic Transaminase [SGOT], Serum Glutamic-Pyruvic Transaminase [SGPT], and Gamma-Glutamyl Transferase [GGT] (and inflammatory markers (C-Reactive Protein [CRP], Tumor Necrosis Factor-alpha [TNF-α], Adiponectin and Irisin). Cinnamon supplement was taken in 500 mg capsules three times a day.

**Results:**

Body mass, BMI, and body fat percentage significantly decreased in all intervention groups (*p* < 0.001), with the greatest fat loss in T + S (−7.86%, *p* < 0.001), significantly more than T (*p* = 0.013). Performance (push-up, squat, plank, jump) improved in T and T + S (all *p* < 0.001), with no difference between them (*p* > 0.05). Fasting blood sugar, insulin, HOMA-IR, and liver enzymes (SGOT, SGPT, GGT) decreased across all interventions (*p* < 0.05), with the greatest reductions in T + S. Inflammatory markers (CRP, TNF-α) declined, while adiponectin and irisin increased in all interventions (*p* < 0.001), with superior changes in T + S versus all groups (*p* < 0.05). The control group showed no significant changes (*p* > 0.05).

**Conclusion:**

Tabata training resulted in synergistically effect on performance, body composition, metabolic-inflammation markers, and liver enzyme function in overweight and obese individuals. Moreover, the cinnamon supplementation as an ergogenic potentiated the observed beneficial effects.

## 1. Introduction

The global rise in overweight and obesity over the past four decades, as highlighted by the World Health Organization, underscores an urgent need for effective intervention strategies [1,2]. This issue is particularly concerning among military personnel, where excess weight can impair physical fitness, health, and operational effectiveness. Lifestyle factors such as prolonged office hours, poor nutrition, lack of sufficient rest, and limited physical activity further exacerbate the risk of cardiovascular and metabolic diseases in this population [3–5].

Obesity-related metabolic alterations are primarily driven by adipokines and myokines, which regulate energy balance and insulin sensitivity through intricate signaling pathways, including autocrine, paracrine, and endocrine mechanisms [6–8]. Among these, tumor necrosis factor-alpha (TNF-α), a pro-inflammatory cytokine secreted by macrophages and monocytes, is closely linked to obesity-related inflammation. Elevated TNF-α levels contribute to insulin resistance, impaired lipid metabolism, and muscle catabolism through inflammatory signaling pathways [9–11].

Conversely, adiponectin, a hormone produced by adipose tissue, enhances glucose uptake and fatty acid oxidation via AMP-activated protein kinase (AMPK) activation, playing a key role in metabolic homeostasis [12–15]. Additionally, the myokine irisin, secreted in response to muscle contraction, promotes white adipose tissue browning and thermogenesis by stimulating mitochondrial biogenesis and upregulating peroxisome proliferator-activated receptor gamma coactivator 1-alpha (PGC-1α) [6,16,17].

Exercise is a well-established intervention for managing obesity and metabolic dysfunction. Both resistance and aerobic training enhance myokine secretion, regulate cytokine release, and improve body composition [18–20]. Among various training modalities, Tabata training, a high-intensity interval protocol consisting of eight cycles of 20 seconds of maximal effort followed by 10 seconds of rest, has gained popularity due to its efficiency in improving cardiovascular fitness, metabolic health, and body composition [21–23]. Research suggests that Tabata training can enhance lipid profiles, insulin sensitivity, and overall metabolic function [24–27].

In addition to exercise, dietary interventions and herbal supplements have been increasingly explored for their potential metabolic benefits. Cinnamon, derived from the bark of Cinnamomum species, has long been recognized for its anti-inflammatory, antioxidant, and blood sugar-regulating properties [28–30]. Studies indicate that cinnamon supplementation may improve insulin sensitivity by enhancing glucose transporter activity and reducing oxidative stress, while also improving lipid profiles and inflammatory markers, making it a promising adjunct to obesity management [31–33].

Given the increasing prevalence of obesity among military personnel, and its strong link to metabolic dysfunction and inflammation, strategies that effectively integrate exercise and nutritional interventions are urgently needed. While Tabata training has demonstrated metabolic and physiological benefits, the potential synergistic effects of combining it with cinnamon supplementation remain unclear. Therefore, this study aims to investigate the effects of an eight-week Tabata training program combined with cinnamon supplementation on metabolic markers, liver enzyme activity, functional performance, and body composition in overweight and obese soldiers.

## 2. Materials and methods

### 2.1. Participants

In this study, 48 overweight and obese soldiers were recruited. Upon expressing their interest, participants were individually provided with a detailed explanation of the study’s objectives and methodology, followed by the completion of a general health and fitness history questionnaire to assess their eligibility. The inclusion criteria required participants to have no prior engagement in regular exercise, maintain a minimum sleep duration of 7–8 hours per day, not use steroids or illicit substances affecting fat oxidation and metabolism in the past year, and have no musculoskeletal disorders. All eligible participants provided written and verbal informed consent before enrollment and completed a medical history questionnaire. The study was conducted following the protocol approved by the Institutional Human Subjects Committee and the Ethics Committee of Islamic Azad University, Sari Branch (IR.IAU.SARI.REC.1403.307), and adhered to the principles outlined in the Declaration of Helsinki.

### 2.2. Study design

**Figure 1.**
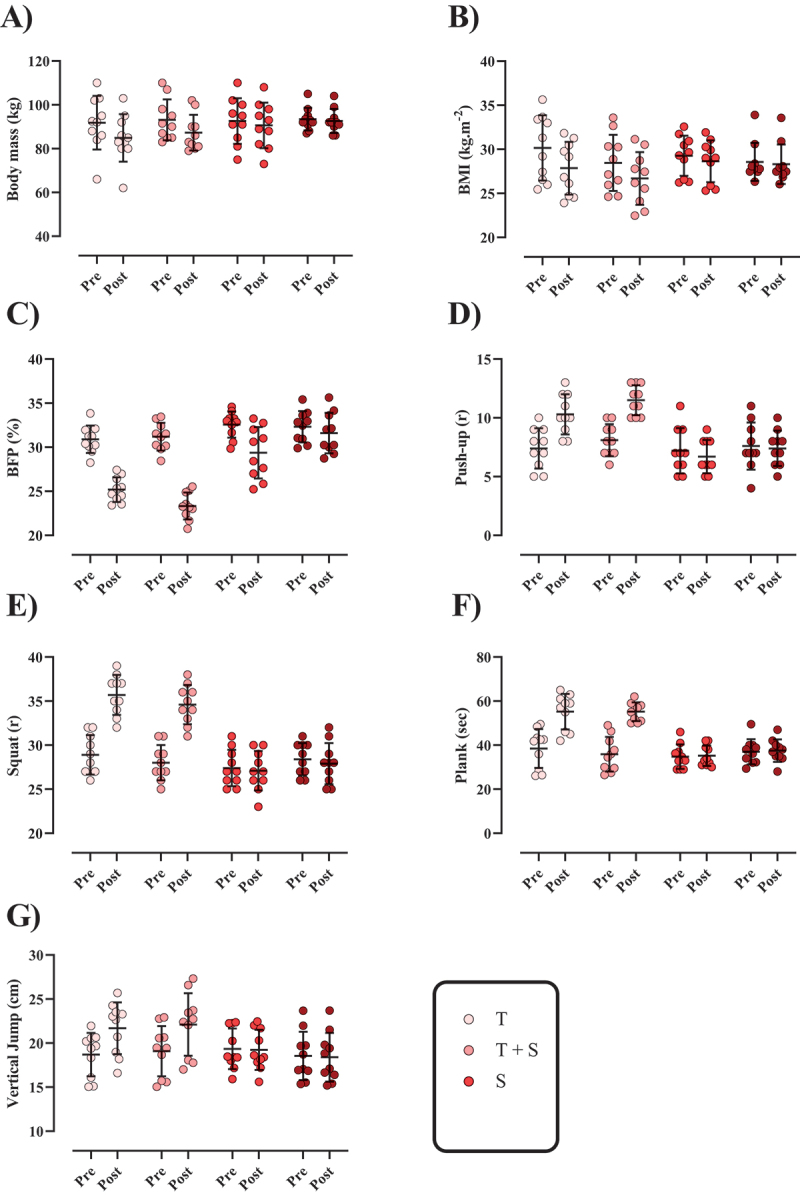
The changes in body composition and performance throughout the intervention. A) body mas (BM), B) body mass index (BMI; [kg.m^−2^]), C) body fat percentage (BFP; [%]), D) push-up (repetition; r), E) squat (repetition; [r]), F) plank (seconds; [sec]), and G) vertical jump (centimeters; [cm]). *N*=10 per group, error bars represent standard deviation (SD), T, Tabata training; T + S, Tabata training + Cinamon supplementation; S, Cinamon supplementation; and C, control.

Figure 1 provides an overview of the study methodology, which utilized a quasi-experimental design, including baseline measurements followed by participant familiarization with the experimental procedures and protocols. Participants were then randomly assigned to one of four groups: Tabata training (T; *n* = 10), Tabata training + cinnamon supplementation (T + S; *n* = 10), cinnamon supplementation (S; *n* = 10), and control (C; *n* = 10). The randomization was conducted using the online platform www.randomizer.org, ensuring unbiased allocation. Over eight weeks, participants underwent a comprehensive exercise intervention. Before beginning the programs, they completed baseline assessments, which included detailed body composition analysis, blood sampling, and functional parameter evaluations. Participants also had a preliminary consultation with the study dietitian to discuss dietary preferences and establish target protein and energy intakes. Sleep quality was assessed using the Pittsburgh Sleep Quality Index (PSQI), and general health status was evaluated through the General Health Questionnaire-28 (GHQ-28), ensuring methodological consistency across all measurement time points [34].

### 2.3. Anthropometric measurements

Using standard anthropometric techniques, the participants’ height and body mass were measured. In this way, height and weight were determined with Seca model 220 measuring device (Germany) and then body mass index (BMI) was calculated. Subcutaneous fat measurements were also taken from the subscapular, triceps, supraspinal, and medial calf skinfolds using a calibrated Harpenden caliper (UK), following the manufacturer’s standardized protocol [35]. After measuring body mass (BM) and body fat percentage (BFP), fat-free mass (FFM) was calculated using the established formula.

(FM = BFP × body mass; FFM = body mass – FM)

### 2.4. Blood sampling

The concentrations of TNF-α, adiponectin, and irisin in the blood were measured using ELISA with kits provided by Eastbiopharm (China). Additionally, liver enzyme levels, including alanine transaminase (ALT), aspartate transaminase (AST), alkaline phosphatase (ALP), gamma-glutamyl transpeptidase (GGT), and C-reactive protein (CRP), were assessed using commercial kits from Delta Darman Part (made in IRAN) according to their standard protocols. Blood samples were collected from participants 48 hours before and after the eight-week intervention, with strict adherence to pre-sampling conditions to ensure accuracy and integrity.

### 2.5. Functional performance test

#### 2.5.1. Push-up test (PUT)

The fitness instructor provided a thorough explanation of the push-up test, ensuring that each participant understood the correct form and positioning. Participants were instructed to position their hands directly under their shoulders, with arms straight and fingers fully extended. Their legs were to remain straight and closely together, with toes firmly planted on the ground. Participants were then asked to lower their bodies by bending their elbows to a 90° angle while maintaining a straight line from their back to their legs. They then continued the movement until their arms were fully extended. A successful execution of this movement was counted as one completed push-up. The test continued until the participant reached their maximum number of repetitions. Each repetition was precisely regulated by a metronome, with a duration of three seconds per movement. The test was concluded if any of the following occurred: voluntary cessation or resting, failure to maintain the correct body position, inability to fully extend the arms, or failure to achieve a 90-degree bend in the elbows during at least two push-ups [36].

#### 2.5.2. Vertical jump test (VJT)

Prior to the test, a skilled expert thoroughly explained the procedure to the participants. They were instructed to stand next to the wall and extend the hand closest to it upwards to its maximum reach. The precise location of their fingertips was then recorded to determine their standing reach height. Following this, participants were asked to jump as high as possible. The vertical distance achieved by the jump was measured by comparing it to the previously recorded standing reach height, yielding the participant’s jump score. The highest score out of three attempts was officially recorded for evaluation [37].

$$\mathrm{Height}\left(\mathrm{cm}\right)\;=\left(g\times \mathrm{tim}e^{2}\right)\;÷\;8\;\;\;g=9.81\;m/s^{2}$$

#### 2.5.3. One-minute sit-to-stand test (STST)

In the one-minute Sit-to-Stand (STS) test, participants were instructed to sit in a standardized chair without armrests, measuring 46 cm in height. They were to ensure their feet were flat on the floor, parallel, and positioned at least hip-width apart, with their arms hanging loosely or resting on their hips. During the test, participants were required to fully extend their knees to achieve a complete standing position. It was essential that their buttocks made contact with the chair when sitting, and the use of arms for assistance was prohibited. Participants were instructed to complete as many sit-to-stand cycles as possible within a 60-second period at their own pace. Only fully completed cycles were counted; incomplete or incorrectly executed movements were not considered. Throughout the test, participants received continuous encouragement to support optimal performance [38].

### 2.6. Tabata training protocol

Tabata workouts are based on two core principles: performing a specific exercise for 20 seconds without interruption, followed by a 10-second rest, and repeating this cycle for a total of eight rounds, resulting in a highly efficient four-minute workout. The training program, conducted over eight weeks, consisted of 24 sessions held three times a week on Saturdays, Mondays, and Wednesdays from 16:00 to 18:00. Each session included a 10-minute warm-up, a 40-minute Tabata protocol, and a 10-minute cool-down, totaling 60 minutes. During the main workout, participants completed eight exercise circuits, each consisting of eight repetitions. Each circuit lasted four minutes, with 20 seconds of intense work followed by 10 seconds of rest, concluding with a one-minute passive rest period. The exercises, performed in a systematic order during each circuit, were: 1. jump squats, 2. back extensions, 3. crunches, 4. push-ups, 5. triceps dips, 6. side crunches, 7. military press with a medicine ball, and 8. chin-ups [27]. All of these exercises were conducted with maximum intensity and controlled by monitoring Heart Rate (Polar, Finland).

### 2.7. Cinnamon supplementation

Participants incorporated 1500 mg of cinnamon powder into their daily diet, consumed three times with meals, over a duration of eight weeks [39].

### 2.8. Diet

Participants were instructed to adhere to their usual dietary habits during the study, submitting three-day food records that encompassed two weekdays and one weekend, both at baseline and following an eight-week intervention; these records were subsequently analyzed with Diet Analysis Plus version 10 to evaluate total energy intake and the contributions from protein, fats, and carbohydrates [40].

### 2.9. Statistical analysis

The a priori sample size calculation was conducted using G*Power 3.1.9.2 software. The rationale for the sample size was based on a previous meta-analysis study, which showed that TNF-α levels were reduced following high-intensity interval training (HIIT). Given the limited number of studies specifically on Tabata, HIIT studies were used as a proxy, as Tabata is a form of HIIT [41]. Based on a small-to-moderate effect size (ES = 0.30), the sample size of 40 participants was calculated to achieve 80% power at a significance level of 0.05. The effect size and sample size estimate suggest adequate statistical power to detect significant differences in TNF-α levels across groups. For the analysis, descriptive statistics (mean and standard deviation) were reported, and the assumptions of normality and homogeneity of variance were checked using Shapiro-Wilk and Levene’s tests, respectively. The analysis of the dependent variables following Tabata training was conducted using Analysis of Covariance (ANCOVA) with SPSS version 26 for statistical evaluation, with significance set at *p* < 0.05.

## 3. Results

### 3.1. Participant characteristics

Eighty participants were assessed for eligibility. Twenty-two did not meet the inclusion criteria, while 10 declined to participate after the first interview. Two participants from each group withdrew due to lack of time and interest. Therefore, a total of 40 participants completed the study. There were no significant between-group differences in baseline characteristics (Table 1).

**Table 1.** Baseline characteristics of the participants.

	T	T+S	S	C
**Measure**				
**Anthropometrics and body composition**				
Age (y)	25 ± 3	25 ± 3	27 ± 3	25 ± 2
Height (cm)	174.5 ± 7.5	181.1 ± 4.79	177 ± 7.2	181 ± 3.97
Body mass (kg)	91.8 ± 12.2	93.1 ± 9.3	92.6 ± 10.3	93.4 ± 5.1
BMI (kg.m^−2^)	30.1 ± 3.6	28.4 ± 3.1	29.2 ± 2.2	28.5 ± 2.1
BFP (%)	30.8 ± 1.5	31.1 ± 1.5	32.5 ± 1.4	32.3 ± 1.7
PSQI (s)	3 ± 1.2	2.7 ± 1.4	3 ± 1.2	2.9 ± 1.1
GHQ-28 (s)	49.6 ± 7.6	50.5 ± 6	49.3 ± 8.8	51.51 ± 7.1
**Performance**				
Push-up (r)	7.4 ± 1.7	8.1 ± 1.3	7.2 ± 1.9	7.6 ± 2
Squat (r)	28.9 ± 2.2	28 ± 2	27.4 ± 2	28.4 ± 1.8
Plank (sec)	38.4 ± 8.7	35.8 ± 7.8	34.7 ± 5.5	37 ± 5.7
Vertical jump (cm)	18.6 ± 2.4	19 ± 2.8	19.3 ± 2.3	18.5 ± 2.7
**Metabolic markers**				
FBS (mg/dl)	104.5 ± 10.3	100.4 ± 4.6	98.1 ± 8.6	101.7 ± 8.8
Insulin (IU)	14.5 ± 3.4	19.4 ± 3.5	17.6 ± 4.7	18 ± 2.9
HOMA	3.7 ± 1.1	4.8 ± 1	4.3 ± 1.4	4.5 ± 0.9
**Liver enzymes**				
SGOT (U/L)	28.8 ± 5.8	24.9 ± 4	29.2 ± 7.1	24.9 ± 4.1
SGPT (U/L)	38.4 ± 12.2	34.9 ± 11.5	34.2 ± 12.7	38.4 ± 9.2
GGT(U/L)	71.8 ± 7.7	68.7 ± 12.6	74.6 ± 10.6	70.8 ± 13.4
**Inflammatory markers**				
CRP (ng/ml)	5.3 ± 1.2	6 ± 1.7	5.8 ± 1.8	4.8 ± 1.5
TNF-α (pg/ml)	143.9 ± 8.9	140.2 ± 16.5	142 ± 10.9	133.7 ± 9
Adiponectin (ng/ml)	16.4 ± 1.2	16.8 ± 1.4	16.2 ± 1.4	16.4 ± 1.4
Irisin (ng/ml)	5.4 ± 0.8	5.8 ± 0.7	5.7 ± 0.6	5.7 ± 0.3

y: Years, cm: Centimeter, kg: Kilograms, body mass index (BMI; [kg.m^−2^]); body fat percentage (BFP; [%]), PSQI: Pittsburgh Sleep Quality Index, GHQ-28: General Health Questionnaire-28, s: Score, push-up (repetition; r), Squat (repetition; [r]), Plank (seconds; [sec]), Vertical Jump (centimeters; [cm]), fasting blood sugar (FBS; [mg/dl]), Insulin (I/U), Homeostatic Model Assessment for Insulin Resistance (HOMA-IR), Serum Glutamic-Oxaloacetic Transaminase (SGOT; [U/I]), Serum Glutamic-Pyruvic Transaminase (SGPT; [U/I]), Gamma-Glutamyl Transferase (GGT; [U/I]); C-Reactive Protein (CRP; [ng/ml]), Tumor Necrosis Factor-alpha (TNF-α; ng/ml), Adiponectin (ng/ml), Irisin (ng/ml); T, Tabata Training; T + S, Tabata Training + Cinamon supplementation; S, Cinamon supplementation; and C, Control.

### 3.2. Adverse events

No adverse events were reported by the participants, or observed by study staff during the testing or training sessions.

### 3.3. Adherence and nutrient intakes

The adherence to training interventions for training groups was 100 %. There were no significant within- or between-group differences for any average daily nutrient or energy intake (*p* > 0.05; Table 2).

**Table 2.** Average dietary intake at pre and post-intervention.

	Time	
	Pre	Post
**Energy (kcal. kg^−1^.d^−1^)**		
T	2477.3 ± 76.7	2506.7 ± 59.6
T + S	2404.5 ± 103.6	2465.7 ± 94.0
S	2420.1 ± 134.9	2417.8 ± 133.7
C	2465.7 ± 137.04	2499.3 ± 113.7
**Protein (g.kg^−1^.d^−1^)**		
T	111.5 ± 10.7	111.3 ± 4.4
T + S	113.8 ± 9.9	110.5 ± 10.4
S	113.7 ± 12.6	119.4 ± 11.9
C	111.0 ± 11.5	109.5 ± 14.4
**Carbohydrate (g.kg^−1^.d^−1^)**		
T	339.8 ± 15.5	352.6 ± 24.0
T + S	344.6 ± 4.6	338.8 ± 18.9
S	342.6 ± 14.2	349.0 ± 16.2
C	342.8 ± 14.2	347.6 ± 24.9
**Fat (g.kg^−1^.d^−1^)**		
T	98.2 ± 9.2	97.9 ± 3.6
T + S	96.7 ± 8.7	94.8 ± 6.8
S	93.4 ± 4.0	96.3 ± 6.3
C	96.0 ± 8.3	98.1 ± 12.7

### 3.4. Body composition

The changes in body composition and anthropometry are shown in Figure 1(A-C). Body mass and BMI significantly decreased in *T* = −6.9 kg (95% CI = −8.5 to −5.31; *p* < 0.001); −2.3 kg·m^− 2^ (95% CI = −2.9 to −1.7, *p* < 0.001), T + S = −5.8 kg (95% CI = −8.25 to −3.44, *p* < 0.001); −1.76 kg·m^− 2^ (95% CI = −2.5 to −1.03, *p* < 0.001), and S = −2 kg (95% CI = −2.47 to −1.52, *p* < 0.001); −0.62 kg·m^− 2^ (95% CI = −0.76 to −0.49, *p* < 0.001) from pre- to post-intervention, while the control group remained unchanged over time (*p* > 0.05). Similar results were observed for BFP in *T* = −5.70 % (95% CI = −6.65 to −4.75; *p* < 0.001); T + S = −7.86 % (95% CI = −8.26 to −7.46, *p* < 0.001), and S = −3.18 % (95% CI = −4.55 to −1.81, *p* = 0.001). The reductions of body mass and BMI in T and T + S groups were significantly greater than other groups (*p* < 0.001) while there was no different between these two groups (*p* > 0.05). Furthermore, no significant difference was observed between the S and C groups (*p* > 0.05). Regarding BFP, a significant difference was found between the T and T + S groups (*p* = 0.013), with the T + S group exhibiting the largest reduction compared to all other groups (*p* < 0.05). Also, the reduction in S group was significantly greater than C group (*p* = 0.003).

### 3.5. Performance

The changes in performance variables are shown in Figure 1(D-G). Push-up [T = 2.9 r (95% CI = 2.67 to 3.12; p < 0.001) and T + S = 5.88 r (95% CI = 5.03 to 6.72, p < 0.001)], squat [T = 6.80 r (95% CI = 6.23 to 7.36; p < 0.001) and T + S = 6.60 r (95% CI = 5.83 to 7.36, p < 0.001)], plank [T = 16.71 s (95% CI = 14.78 to 18.63; p < 0.001) and T + S = 19.31 s (95% CI = 14.96 to 23.65, p < 0.001)], and vertical jump height [T = 2.99 cm (95% CI = 2.48 to 3.49; p < 0.001) and T + S = 3.02 cm (95% CI = 2.47 to 3.57, p < 0.001)] significantly increased from pre to post, while the C group remained unchanged (p > 0.05). There was no significant difference between T and T + S groups (p > 0.05).

### 3.6. Metabolic markers

**Figure 2.**
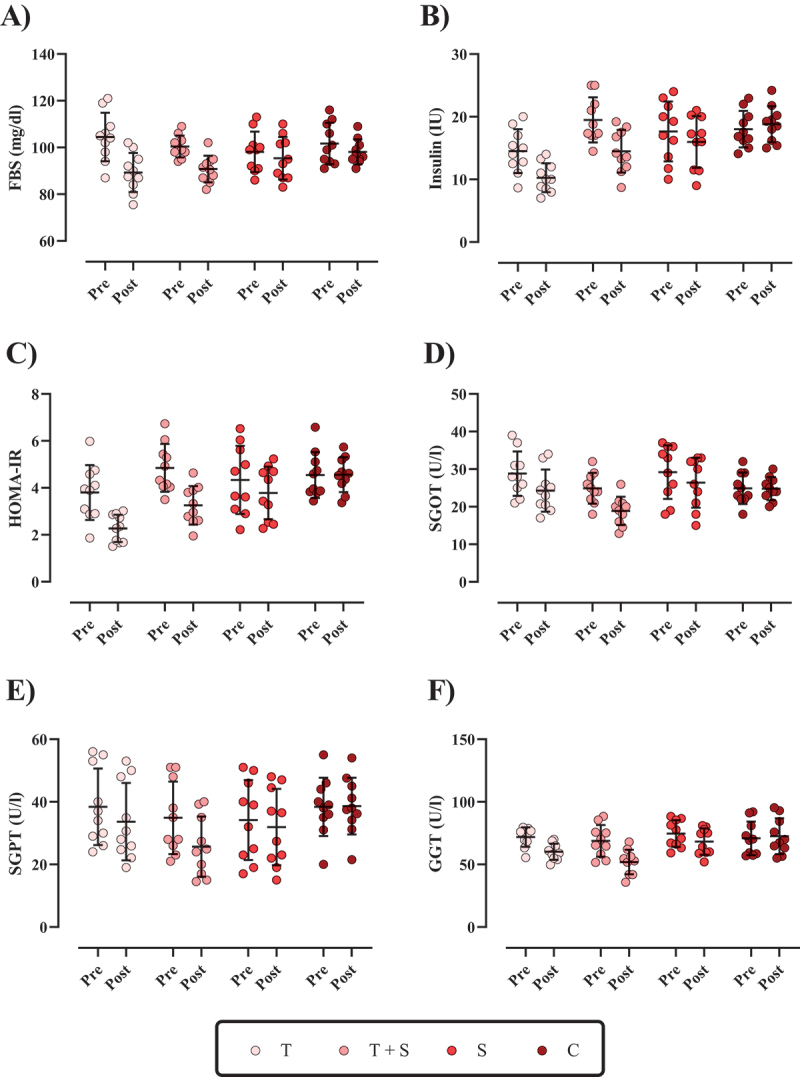
The changes in metabolic markers and liver enzymes throughout the intervention. A) fasting blood sugar (FBS; [mg/dl]), B) insulin (I/U), C) homeostatic model assessment for insulin resistance (HOMA-IR), D) serum Glutamic-Oxaloacetic Transaminase (SGOT; [U/I]), E) serum Glutamic-Pyruvic Transaminase (SGPT; [U/I]), and F) gamma-Glutamyl Transferase (GGT; [U/I]). *N*=10 per group, error bars represent standard deviation (SD), T, Tabata training; T + S, Tabata training + Cinamon supplementation; S, Cinamon supplementation; and C, control.

The changes in metabolic markers are shown in Figure 2(A-C). FBS [*T* = −15.2 mg/dl (95% CI = −20.25 to −10.14; *p* < 0.001); T + S = −9.6 mg/dl (95% CI = −11.57 to −7.62, *p* < 0.001), and S = −2.75 mg/dl (95% CI = −4.41 to −1.08, *p* = 0.005)], insulin [*T* = −4.25 IU (95% CI = −5.89 to −2.61; *p* < 0.001); T + S = −5 IU (95% CI = −5.73 to −4.27, *p* < 0.001), and S = −1.67 IU (95% CI = −3.03 to −0.28, *p* = 0.023)], and HOMA [*T* = −1.52 (95% CI = −2.06 to −0.98; *p* < 0.001); T + S = −1.59 (95% CI = −1.84 to −1.35, *p* < 0.001), and S = −0.55 (95% CI = −0.91 to −0.20, *p* = 0.006)] significantly decreased from pre to post-intervention in all three interventions, while the control group remained unchanged (*p* > 0.05). Regarding all metabolic markers, the reductions in T + S were significantly greater than S and C, T than S and C. Moreover, there was a significant difference between C and S, except for FBS.

### 3.7. Liver enzymes

The changes in liver enzymes are shown in Figure 2(D-F). SGOT [T = −4.51 U/L (95% CI = −5.58 to −3.44; p < 0.001); T + S = −6.01 U/L (95% CI = −7.01 to −5.01, p < 0.001), and S = −2.80 U/L (95% CI = −3.45 to −2.14, p < 0.001)], SGPT [T = −4.75 U/L (95% CI = −5.46 to −4.04; p < 0.001); T + S = −9.19 U/L (95% CI = −11.34 to −7.04, p < 0.001), and S = −2.25 U/L (95% CI = −3.28 to −1.22, p = 0.001)], and GGT [T = −11.70 U/L (95% CI = −15.90 to −7.50; p < 0.001); T + S = −16.86 U/L (95% CI = −20.46 to −13.26, p < 0.001), and S = −6.33 U/L (95% CI = −7.53 to −5.14, p < 0.001)] significantly decreased from pre to post-intervention in all three interventions, while the control group remained unchanged (p > 0.05). For all liver enzymes, the reductions in T + S were significantly greater than all groups, T than S and C, and S than C.

### 3.8. Inflammatory markers

**Figure 3.**
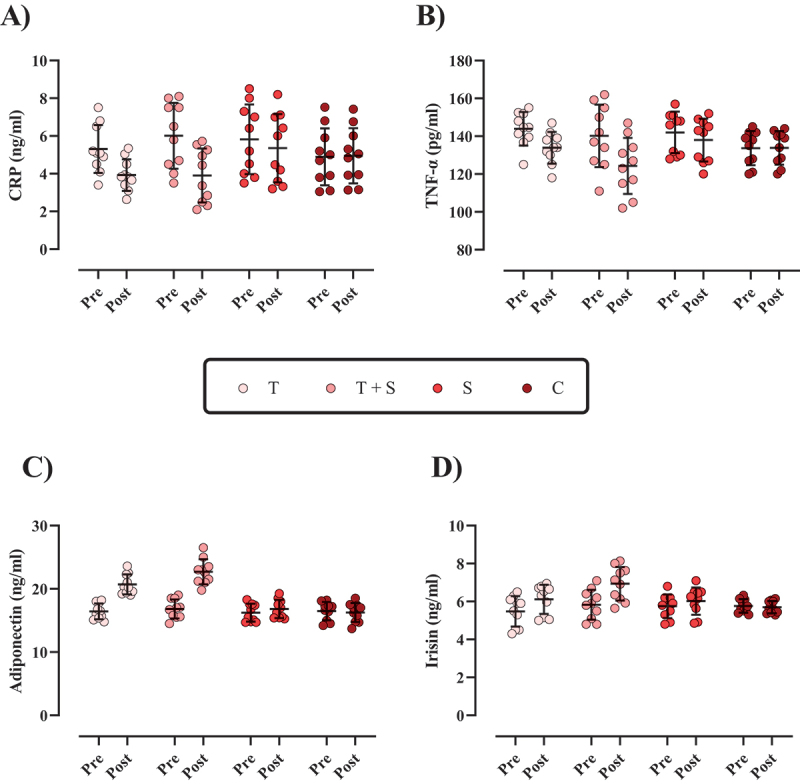
The changes in inflammatory markers throughout the intervention. A) C-Reactive protein (CRP; [ng/ml]), B) tumor necrosis factor-alpha (TNF-α; ng/ml), C) adiponectin (ng/ml), and D) irisin (ng/ml). *N*=10 per group, error bars represent standard deviation (SD), T, Tabata training; T + S, Tabata training + Cinamon supplementation; S, Cinamon supplementation; and C, control.

The changes in inflammatory markers are shown in Figure 3(A-D). CRP [T = −1.38 ng/ml (95% CI = −1.84 to −0.92; p < 0.001); T + S = −2.10 ng/ml (95% CI = −2.52 to −1.68, p < 0.001), and S = −0.46 ng/ml (95% CI = −0.61 to −0.32, p < 0.001)] and TNF-α [T = −10.05 pg/ml (95% CI = −12.01 to −8.08; p < 0.001); T + S = −15.92 pg/ml (95% CI = −22.26 to −9.57, p < 0.001), and S = −4 pg/ml (95% CI = −5.21 to −2.78, p < 0.000)] were significantly reduced from pre to post-intervention, while adiponectin [T = 4.28 ng/ml (95% CI = 3.90 to 4.65; p < 0.001); T + S = 5.88 ng/ml (95% CI = 5.03 to 6.72, p < 0.001), and S = 0.58 ng/ml (95% CI = 0.33 to 0.82, p < 0.001)] and irisin [T = 0.63 ng/ml (95% CI = 0.54 to 0.72; p < 0.001); T + S = 1.10 ng/ml (95% CI = 0.89 to 1.32, p < 0.001), and S = 0.26 ng/ml (95% CI = 0.17 to 0.36, p < 0.001)] were significantly increased from pre to post. However, the C group remained unchanged over time for all markers (p > 0.05). For all markers, the changes in T + S were significantly greater than those in all other groups, and the changes in T were significantly greater than in S and C.

## 4. Discussion

This study explored the combined effects of Tabata training and cinnamon supplementation on metabolic markers, liver enzymes, functional performance, and body composition in overweight and obese soldiers. The key findings indicate that Tabata training alone significantly improved cytokines such as adiponectin, leptin, and the myokine irisin. This intervention also enhanced liver enzyme profiles, insulin resistance, and functional capacity. Importantly, the combination of Tabata training and cinnamon supplementation led to greater improvements across multiple health metrics, including reduced body fat percentage, improved insulin sensitivity, healthier liver enzyme levels, and lower inflammatory markers compared to non-combined interventions. The rise in irisin further highlights the intervention’s role in enhancing metabolic health.

### 4.1. Body composition

Body composition parameters, including body mass, BMI, and BFP showed significant reductions in both the T and T+C groups compared to the other two groups. Notably, BFP reduction was greater in the T+C group than in the T group, although changes in body mass and BMI did not differ significantly between these two groups. These findings are consistent with previous studies demonstrating the effectiveness of Tabata training in improving body composition. Doramazki et al. (2020) reported significant reductions in body mass, waist-to-hip ratio, and BFP following ten weeks of Tabata training with the bioelectrical impedance method [25]. Similarly, a 16-week intervention in inactive middle-aged women led to substantial decreases in fat mass, BMI, and upper limb and wrist circumferences compared to a control group Using the Bioelectrical Impedance Analysis (BIA) technique for body composition assessment [42]. Trough bioelectrical impedance analysis, Lu et al. (2023) also found reductions in fat mass, BMI, and waist circumference after 12 weeks of Tabata training in young female students [43]. Existing evidence supports Tabata training as an effective approach for enhancing body composition in sedentary populations [44,45]. As a form of HIIT, Tabata may promote physiological adaptations that increase post-exercise fatty acid metabolism and oxidation, potentially through mechanisms involving catecholamines, growth hormone, and mitochondrial biogenesis mediated by AMPK – PGC-1α signaling. The results of the current study further confirm that short-duration, high-intensity exercise can be effective for improving body composition, consistent with findings from other Tabata-based interventions [27,43,46]. Furthermore, cinnamon supplementation (500 mg, three times daily) may synergistically enhance fat metabolism when combined with Tabata training. Compounds such as methyl hydroxychalcone polymers, flavonoids, and phenolic complexes in cinnamon may facilitate fat cell metabolism by modulating blood glucose and insulin homeostasis [47]. Additionally, cinnamon may improve metabolic function by promoting the expression of tristetraprolin, an anti-inflammatory protein often downregulated in overweight and obese individuals [48]. In conclusion, Tabata training significantly improved body composition, and these effects were further enhanced through daily cinnamon supplementation, suggesting a potentially synergistic intervention for overweight and obese populations.

### 4.2. Performance

The T and T+C groups demonstrated similarly significant improvements in performance measures, including push-ups, squats, and plank duration, relative to the control and supplement-only groups. Current evidence suggests that Tabata training can meaningfully enhance functional abilities and physical performance across various populations [27,43,49–51]. Amabardi et al. (2023) reported that as few as three weekly Tabata sessions elicited physical adaptations comparable to or in some cases exceeding those induced by standard military training. They argued that Tabata training more efficiently supports functional adaptations and improves quality of life in military personnel [49]. Lu et al. (2023) similarly observed that a 12-week low-volume Tabata-style functional HIIT program significantly improved cardiorespiratory fitness in female university students [43]. In trained runners, Tabata training has also been shown to enhance pulmonary function, endurance, and race performance, suggesting superior efficiency relative to traditional training approaches [50]. However, not all findings have been consistent. Dermazsky et al. (2020) found that 10 weeks of Tabata-style HIIT did not lead to significant improvements in aerobic capacity, agility, or explosive power among adolescents [25]. Such discrepancies likely reflect differences in study populations, training protocols, and baseline fitness levels. The current study demonstrated that eight weeks of Tabata training, incorporating 20-second high-intensity intervals with 10-second rest periods, significantly improved endurance and muscle strength. These enhancements may be attributed to neuromuscular and metabolic adaptations elicited by the training [43,52]. Tabata training promotes both anaerobic and aerobic energy system development, contributing to improvements in strength, endurance, and motor skill efficiency [27,51].

While cinnamon supplementation is known for its anti-inflammatory properties that may facilitate recovery, its combination with Tabata training did not result in statistically significant additional improvements in performance outcomes in this study. Nonetheless, emerging evidence suggests that cinnamon may influence longer-term physiological adaptations and potentially reduce the incidence or severity of delayed onset muscle soreness [53]. Overall, Tabata training effectively improved functional performance. However, the addition of cinnamon supplementation did not significantly amplify these improvements beyond those observed with training alone.

### 4.3. Metabolic markers

Significant reductions in FBS, insulin, and HOMA-IR were observed in both the T and T+C groups compared to the C and C-only groups. Moreover, these reductions were more pronounced in the T+C group relative to the T group alone.

Tabata training has been shown to elicit higher rates of carbohydrate metabolism than moderate-intensity exercise during both exercise and recovery phases, thereby improving glycemic control [54]. While Lu et al. (2023) reported no significant changes in fasting glucose or insulin, they did observe a significant reduction in insulin resistance following a 12-week Tabata program [43]. Multiple studies support the notion that HIIT can enhance carbohydrate metabolism and improve insulin sensitivity [55–57]. For instance, Matos et al. (2018) found that HIIT improved oxidative metabolism and reduced insulin resistance in obese individuals [55], and Jelleyman et al. (2015) confirmed that HIIT significantly decreases insulin resistance, particularly in individuals with elevated fasting glucose or type 2 diabetes [56]. However, not all studies report consistent results. Arad et al. (2015) found no significant changes in insulin sensitivity following a 14-week HIIT protocol in overweight and obese women, potentially due to gender-specific physiological responses [58]. The observed metabolic adaptations may be mediated by AMPK, a key energy sensor activated during high-intensity exercise, which stimulates glucose uptake by promoting GLUT4 (Glucose Transporter Type 4) translocation to the cell membrane [59,60]. Additionally, elevated epinephrine levels during exercise may support insulin-independent glucose uptake, while anti-inflammatory myokines like interleukin-6 (IL-6) may enhance insulin receptor sensitivity and downstream signaling [61,62].

In the current study, cinnamon supplementation further amplified the metabolic benefits of Tabata training. Cinnamon extract has been shown to activate insulin receptors and downstream signaling cascades while increasing GLUT expression in adipocytes [63]. Moreover, animal studies indicate that cinnamon modulates gluconeogenesis via the AMPKα/PGC-1α axis by enhancing AMPKα phosphorylation and upregulating sirtuin-1 (SIRT-1) and PGC-1α expression, leading to suppressed hepatic glucose production [64]. Therefore, the combination of cinnamon supplementation and Tabata training appears to synergistically enhance carbohydrate metabolism and improve insulin sensitivity in overweight and obese individuals.

### 4.4. Liver enzymes

The findings revealed a marked reduction in liver enzyme levels (AST, ALT, and GGT) in the T+C group compared to all other groups. The T group also demonstrated a significant decline relative to the supplement-only and control groups. Variations in AST, ALT, and GGT levels reflect liver tissue integrity and function [65]. Although no studies have specifically evaluated the effect of Tabata training on liver enzymes, research on HIIT of which Tabata is a subtype has demonstrated its potential to influence hepatic enzyme levels. A recent study reported that nine weeks of HIIT significantly improved liver enzyme profiles in overweight/obese adolescent girls, suggesting its effectiveness in addressing metabolic dysfunction-associated steatotic liver disease (MASLD) and its complications [66]. Similarly, Hemmatinafar et al. (2020) found that eight weeks of HIIT led to favorable changes in liver enzymes in obese young men [67]. A meta-analysis further confirmed that HIIT significantly lowers ALT and AST levels in individuals with MASLD [68]. However, some studies have reported no significant changes in liver enzymes following HIIT interventions. For example, after a six-week program in healthy young adults or a 12-week regimen in individuals with type 2 diabetes [69,70]. These discrepancies may be attributed to differences in participant characteristics, training duration, and exercise intensity across studies. The current findings suggest that Tabata training alone can improve liver enzyme profiles, potentially through mechanisms associated with AMPK activation. During intense exercise, AMPK plays a crucial role in enhancing insulin sensitivity and reducing hepatic lipid accumulation by promoting fatty acid oxidation and suppressing lipogenesis in hepatocytes [71–73]. Additionally, HIIT has been shown to support liver function by enhancing antioxidant capacity [74], promoting mitochondrial biogenesis [60], stimulating the release of anti-inflammatory cytokines [75], and increasing fatty acid oxidation [76]. However, several studies have investigated the synergistic effects of cinnamon supplementation in conjunction with structured exercise regimens [77–79]. Cinnamon, when utilized as an herbal supplement, demonstrated a notable improvement in liver enzyme function in conjunction with Tabata training. Several randomized controlled trials (RCTs) have demonstrated a modest reduction in ALT, ALP, and AST levels with cinnamon supplementation [80], though conflicting evidence from other studies suggests no significant clinical benefit [81]. The inherent properties of cinnamon, including cinnamaldehyde, cinnamic acid, and polyphenols, contribute significantly to enhancing liver metabolic function [82]. While the exact mechanisms remain unclear, cinnamon has been proposed to act via the activation of peroxisome proliferator-activated receptor gamma (PPARγ), which may improve liver enzyme function and downregulate inflammatory cytokines [83]. Therefore, cinnamon supplementation, when combined with Tabata training, may offer an effective strategy for modulating liver enzyme activity and supporting liver health in overweight and obese individuals.

### 4.5. Inflammatory markers

The results revealed that the T+C group exhibited the most pronounced reduction in inflammatory markers and the greatest increase in anti-inflammatory markers relative to all other groups. However, significant improvements were also observed in both the T-only and C-only groups compared to the control group. These findings suggest that both Tabata training and cinnamon supplementation independently confer anti-inflammatory benefits, while their combination appears to exert synergistic effects.

Murawska-Ciałowicz et al. (2020) reported that eight weeks of Tabata training significantly increased circulating irisin levels in men [27]. Irisin, a short-lived myokine cleaved from fibronectin type III domain-containing protein 5 (FNDC5) and regulated by PGC-1α, plays a key role in mediating the anti-inflammatory and metabolic benefits of exercise [84]. Multiple studies have demonstrated that HIIT effectively elevates irisin [85] and adiponectin levels [86] while concurrently reducing inflammatory markers such as CRP [87] and TNF-α [88]. The reduction in inflammatory cytokines may be partially attributed to the loss of visceral adipose tissue, a primary source of pro-inflammatory cytokines like TNF-α. Exercise-induced fat loss has been shown to reduce TNF-α and simultaneously increase adiponectin, thereby enhancing its anti-inflammatory actions [86,89–91]. Elevated adiponectin promotes fatty acid oxidation via activation of AMPK and modulation of peroxisome proliferator-activated receptor alpha (PPAR-α) activity [92]. Moreover, chronic exercise adaptations have been associated with reduced resting CRP levels, achieved through various mechanisms such as decreased cytokine production from adipose tissue, enhanced skeletal muscle myokine secretion, improved endothelial function and insulin sensitivity, and elevated antioxidant capacity [93–95].

In addition, cinnamon supplementation, whether administered independently or alongside Tabata training, further enhanced anti-inflammatory responses. Several studies have shown that cinnamon intake in conjunction with regular exercise results in a decrease in pro-inflammatory cytokines and a concurrent increase in anti-inflammatory markers [78,96]. Cinnamon’s bioactive compounds, particularly cinnamaldehyde, have been shown to modulate immune function by influencing the expression of genes involved in both pro- and anti-inflammatory processes [33,97–99]. Furthermore, flavonoids extracted from cinnamon such as hesperidin and quercetin possess potent anti-inflammatory properties [100,101]. Numerous studies have confirmed that cinnamon extract and its polyphenols reduce serum concentrations of TNF-α, CRP, and IL-6, improve clinical symptoms, and enhance antioxidant activity [102,103].

### 4.6. Limitations

The current study has several limitations. A limitation of this study was the absence of a placebo- group. A further limitation of this study was the absence of measurements of molecular pathways; additional research is therefore necessary to elucidate these mechanisms.

The absence of lean and fat mass measurements using dual-energy X-ray absorptiometry (DXA) raises concerns about the internal validity of the findings, particularly since many participants had prior training experience but were unfamiliar with Tabata training. Additionally, the brief duration of both the training regimen and cinnamon supplementation limits our ability to determine whether the observed effects would persist or become more pronounced with a longer intervention period. While perceived exertion (RPE) was used to control exercise intensity, future studies should consider employing heart rate monitoring for more precise and objective regulation of exercise load. Finally, the demanding nature of the Tabata training protocol may be too intense for individuals with lower fitness levels, particularly those classified as overweight or obese, and future investigations should explore adjustments to the training protocol to improve its applicability for these populations.

## 5. Conclusion

Overall, participating in Tabata as an intense intermittent exercise regimen significantly improved both inflammatory and anti-inflammatory markers in overweight and obese individuals. This was accompanied by reductions in fat and body weight, leading to substantial improvements in functional capacity within a relatively short period.
